# Radiologic Features of Curvilinear and Tubulonodular Pericallosal Lipomas: Two Case Reports

**DOI:** 10.7759/cureus.99319

**Published:** 2025-12-15

**Authors:** Abdelwahed Diani, Fatima-Ezzahra Akhatar, Mohammed reda Bouroumane, Meriam Benzalim, Soumaya Alj

**Affiliations:** 1 Radiology, Ibn Tofail Hospital, Mohamed VI University Hospital, Faculty of Medicine and Pharmacy, Cadi Ayyad University, Marrakech, MAR; 2 Radiology, Mohamed VI University Hospital, Faculty of Medicine and Pharmacy, Cadi Ayyad University, Marrakech, MAR

**Keywords:** corpus callosum, curvilinear, mri, pericallosal lipoma, tubulonodular

## Abstract

Pericallosal lipomas or lipomas of the corpus callosum are rare, benign, fat-containing congenital brain lesions. They may occur in isolation or be associated with corpus callosum dysgenesis or agenesis. Therefore, they have a broad clinical presentation, ranging from being totally asymptomatic to having seizures, motor deficits, or headaches. Imaging techniques such as computed tomography (CT) and magnetic resonance imaging (MRI) are key to diagnosis, allowing recognition of two morphological subtypes: tubulonodular and curvilinear.

In this article, we report two cases. The first concerns a 35-year-old woman evaluated for helmet-like headaches, in whom a brain MRI demonstrated a curvilinear lipoma of the corpus callosum. The second case concerns a 33-year-old man with headaches following a benign trauma, in whom CT and MRI identified a tubulonodular corpus callosum lipoma. These cases emphasize the significance of imaging in distinguishing between these two forms.

## Introduction

Intracranial lipomas are uncommon, accounting for 0.06-0.46% of all intracranial lesions.They typically occur along the supratentorial midline, particularly within the pericallosal cistern, and are therefore called pericallosal lipomas [1].

Intracranial lipomas are now recognized as congenital malformations resulting from the differentiation of persistent primitive meninges into adipose tissue [1]. Two morphological types of intracranial lipomas have been identified: tubulonodular and curvilinear. These lipomas are often associated with agenesis or hypogenesis of the corpus callosum. Corpus callosum lipomas are typically asymptomatic and are most often discovered incidentally during imaging performed for other conditions, frequently after trauma, with growth occurring at a very slow rate [2].

We report two cases: a 35-year-old woman with helmet-like headaches, diagnosed with a curvilinear corpus callosum lipoma on MRI, and a 33-year-old man who developed headaches after mild trauma, with computed tomography (CT) and magnetic resonance imaging (MRI) revealing a tubulonodular corpus callosum lipoma.

## Case presentation

Case 1

A 35-year-old female patient with no significant medical history presented with helmet-like headaches. Her vital signs were normal, and the physical examination revealed no abnormalities or focal neurological deficits. 

**Figure 1:**
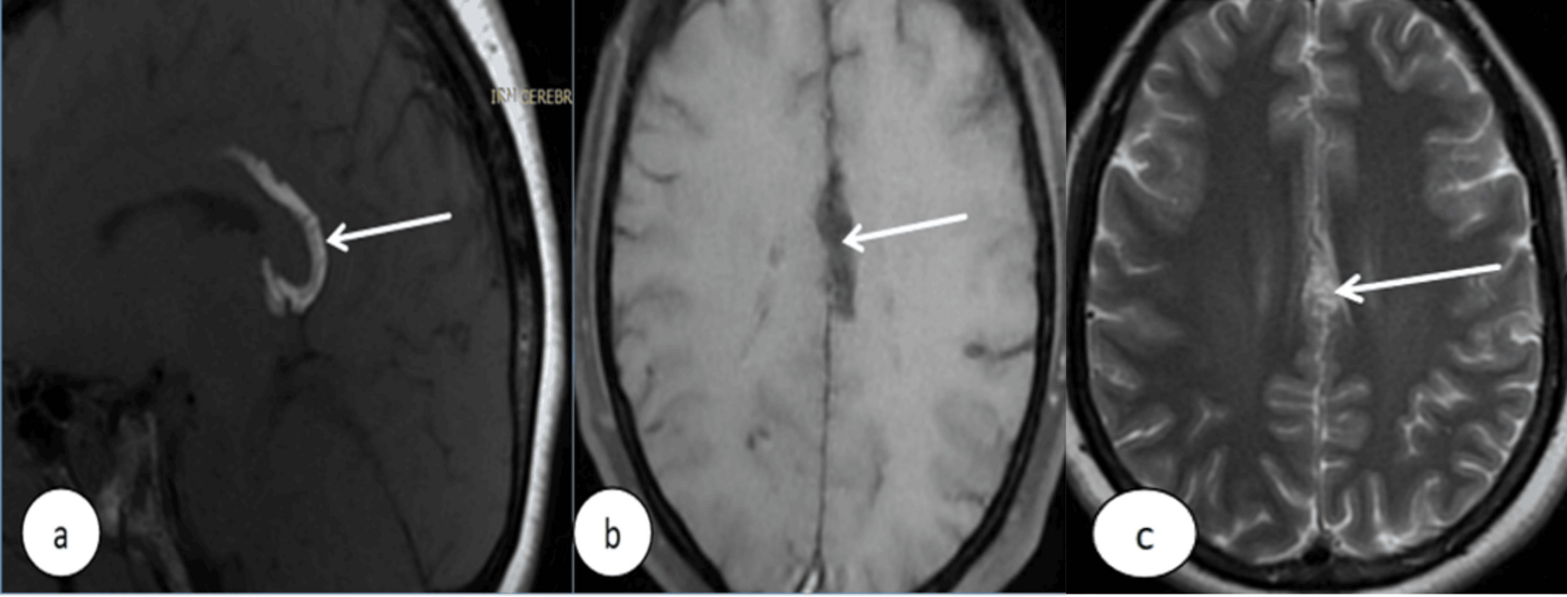
Brain MRI with (a) sagittal T1 FSE, (b) axial T1 Fat Saturation, and (c) axial T2 showing a curvilinear pericallosal lipoma (arrows), appearing hyperintense on T1 and T2, with signal loss on the T1 Fat Saturation sequence. MRI: magnetic resonance imaging (MRI); T1 FSE: fast spin echo; T1 Fat Sat: MRI T1 fat saturated

The patient was referred to our radiology department for further evaluation via brain imaging. MRI revealed a curvilinear pericallosal lipoma adjacent to the splenium of the corpus callosum, measuring approximately 8 mm in thickness and 72 mm in length. Distinct calcifications were present, appearing as signal voids on the T2 sequence (Figure 1).

Both the rostrum and splenium appeared intact, confirming the absence of callosal dysgenesis. Based on these findings, a diagnosis of pericallosal curvilinear lipoma was established, and no surgical treatment was deemed necessary. The patient was managed symptomatically.

Case 2

**Figure 2:**
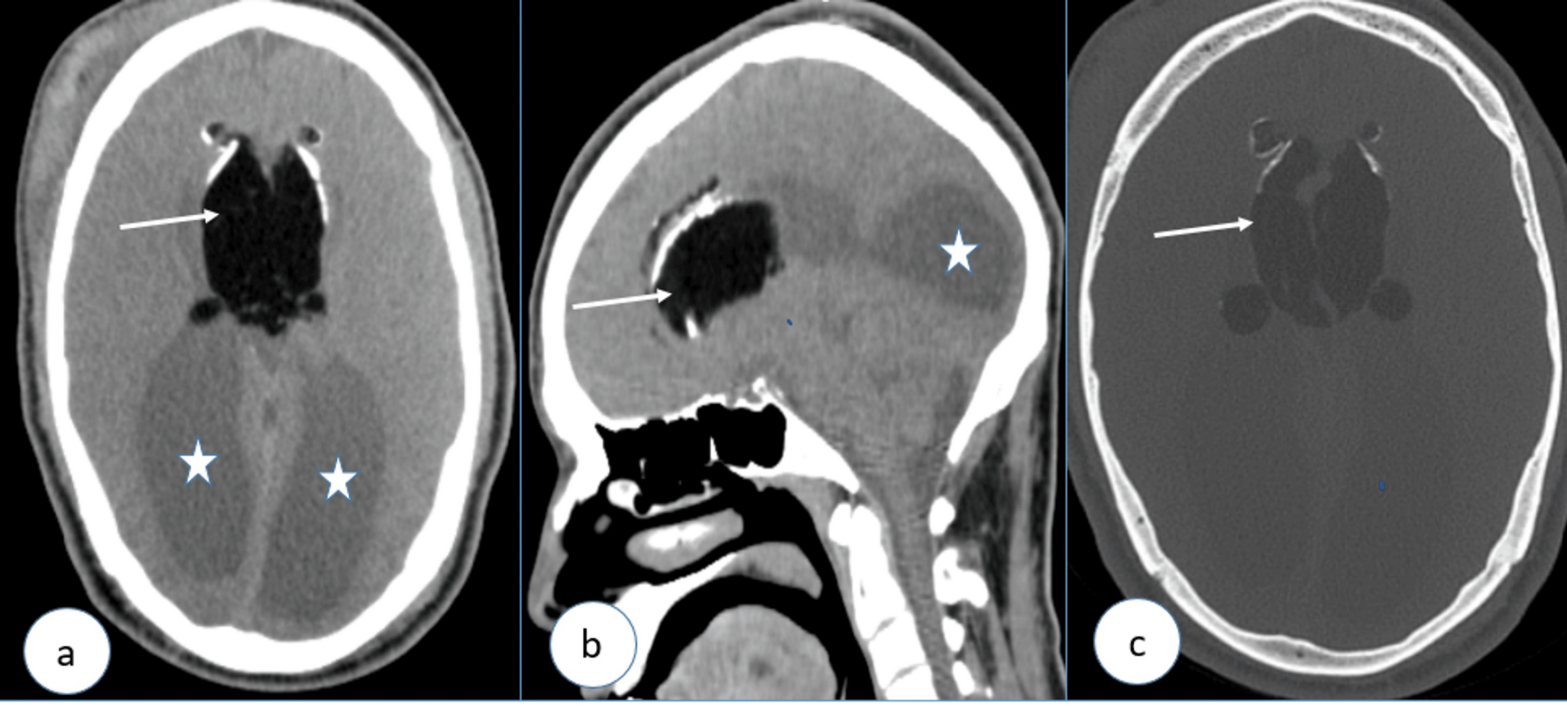
Brain CT in parenchymal window with axial (a) and sagittal (b) views, and in bone window (c), showing an intra-axial fatty lesion with peripheral calcifications (white arrows) located in the interhemispheric fissure and centered on the corpus callosum. The ventricles are widely spaced with colpocephaly (stars) in keeping with corpus callosal dysgenesis CT: computed tomography

A 33-year-old patient with a history of mild head trauma consulted for headaches. The general and neurological examinations were unremarkable, except for a frontal scalp hematoma, which was treated medically. Routine laboratory tests were within normal limits. A brain CT scan revealed a well-defined intra-axial fatty lesion with peripheral calcifications, centered on the anterior portion of the corpus callosum. T“The lesion measured 37 mm in thickness and 56 mm in length and caused displacement of the frontal horns of the lateral ventricles, extending intraventricularly through the foramina of Monro in a bilateral and symmetrical manner (Figure 2).

**Figure 3:**
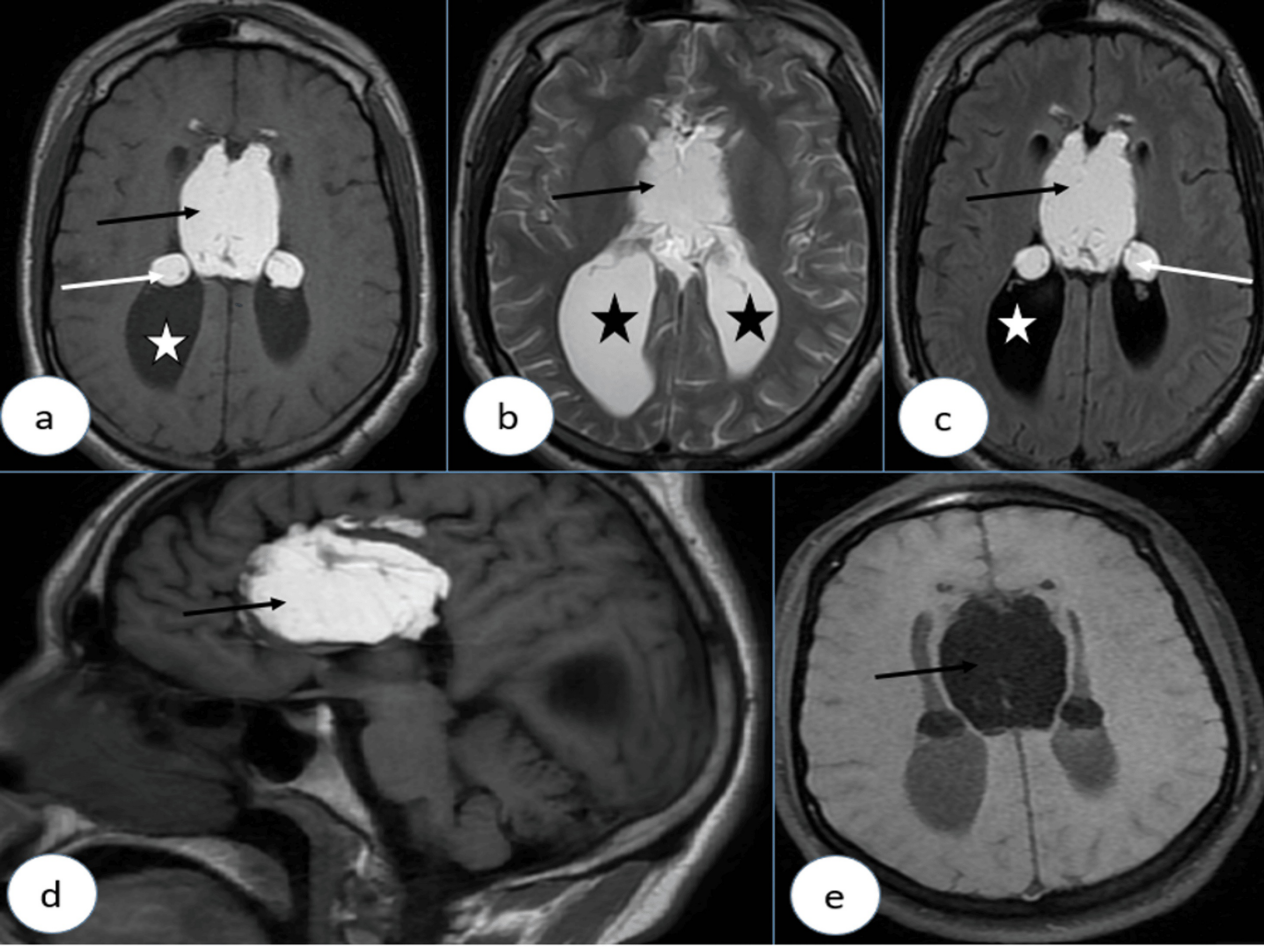
Brain MRI with axial T1 FSE (A), axial T2 (B), axial T2 FLAIR (C), sagittal T1 FSE (D) and axial T1 Fat Sat (E), showing a tubulonodular pericallosal lipoma (black arrows), appearing hyperintense on T1 and T2, with signal loss on the T1 Fat Saturation sequence, associated with a agenesis of the splenium of the corpus callosum and colpocephaly (stars). Intraventricular extension through the foramina of Monro (white arrows) is also noted MRI: magnetic resonance imaging (MRI); T1 FSE: fast spin echo; T1 Fat Sat: MRI T1 fat saturated; FLAIR: fluid-attenuated inversion recovery

MRI confirmed these findings, showing a lesion centered on the body and genu of the corpus callosum, appearing hyperintense on T1, T2, and FLAIR sequences, with signal suppression on T1 Fat Saturation and no diffusion restriction. Additionally, colpocephaly was noted, associated with agenesis of the splenium of the corpus callosum. These imaging features were consistent with a nodular form of corpus callosum lipoma (Figure 3).

Similar to the prior case, management was symptomatic, and no surgical intervention was required.

## Discussion

Intracranial lipomas are extremely rare congenital malformations, accounting for less than 0.1% of intracranial tumors [3]. They result from the abnormal differentiation of the persistent meninx primitiva, a region that forms the inner layer of the pia-arachnoid and dura mater [2].

Historically, Meckel first described a chiasmatic lipoma in 1818, and in 1856, Rokitansky provided the initial description of a pericallosal lipoma associated with agenesis of the corpus callosum [4]. Pericallosal lipomas are classified into two groups: tubulonodular lipomas, which are large, anteriorly located, and associated with brain anomalies like corpus callosum dysgenesis, and curvilinear lipomas, which are smaller, posteriorly located, and usually have a normal corpus callosum with fewer and less severe associated anomalies [5]. Hypogenesis or agenesis of the corpus callosum is frequently associated with pericallosal lipomas, occurring in approximately 90% of anterior lipomas and 30% of posterior lipomas [5]. 

Clinically, an isolated lesion of the corpus callosum is unlikely to produce symptoms [6], and approximately half of cases are discovered incidentally. When present, symptoms may include headaches, seizures, and limb weakness, and patients often seek medical attention for psychological complaints and memory problems [7,8]. Clinical manifestations, such as seizures and mental disorders, should be attributed to associated neurological tissue abnormalities [9]. Epilepsy is among the most common symptoms, typically manifesting before the age of 15 and often presenting as partial, severe seizures [10-11].

Cranial CT and MRI provide highly characteristic findings that aid in the diagnosis of pericallosal lipomas. CT shows a well-defined, midline pericallosal mass that is homogeneous and hypodense, with a density range of -40 UH to -100 UH, and may include peripheral calcifications [12]. MRI is particularly valuable for assessing the extent of the lipoma by identifying associated corpus callosum abnormalities and ruling out differential diagnoses, especially intracranial teratomas. These tumors exhibit fat-like attenuation characteristics, appearing hyperintense on T1 and T2 sequences, showing signal attenuation on fat-suppressed sequences, and do not enhance after gadolinium injection [12].

Differential diagnoses are generally limited and may include intracranial teratoma, fat within the cerebral falx, or lipomatous transformation of tumors such as gliomas, ependymomas, and primitive neuroectodermal tumors [13].

Surgical removal of an intracranial lipoma is risky due to the high mortality and morbidity associated with its attachment to surrounding tissues and neurovascular structures. “Although lipomas generally do not require surgical intervention, surgery may be considered in cases of uncontrolled seizures, hydrocephalus, progressive dementia, or elevated intracranial pressure [7-8]. Rather than total excision, the surgical approach should aim for decompression. In our case, there were no surgical indications, and the patient was managed with clinical follow-up.

## Conclusions

Pericallosal lipoma is a rare, benign intracranial tumor that is typically asymptomatic. Diagnosis is primarily made through CT or MR imaging, with MRI being the modality of choice. Imaging helps differentiate between the two types: tubulonodular and curvilinear. Surgical intervention is infrequent, and the prognosis is generally favorable, depending on the presence of associated malformations.
